# Artificial intelligence assessment of valvular disease and ventricular function by a single echocardiography view

**DOI:** 10.3389/fdgth.2025.1684933

**Published:** 2026-01-12

**Authors:** Lior Fisher, Michael Fiman, Ella Segal, Shira Lidar, Noa Rubin, Adiel Am-Shalom, Ido Cohen, Kobi Faierstein, Avishai M. Tsur, Ehud Schwammenthal, Robert Klempfner, Eyal Zimlichman, Ehud Raanani, Elad Maor

**Affiliations:** 1 Sheba Medical Center, Leviev Cardiovascular Institute, Ramat Gan, Israel; 2 Sheba Medical Center, Tel-Aviv Faculty of Medicine, Tel Aviv University, Tel Aviv, Israel; 3 Department of Military Medicine, Faculty of Medicine, Hebrew University of Jerusalem, Jerusalem, Israel; 4 School of Public Health, Gray Faculty of Medical & Health Sciences, Tel Aviv University, Tel Aviv, Israel

**Keywords:** artificial intelligence, heart failure, echocardiography, point-of-care imaging, valvular heart disease

## Abstract

**Background:**

Valvular heart disease and heart failure are major global health burdens, yet access to comprehensive echocardiography is often limited, particularly in resource-constrained settings. Artificial intelligence (AI) may enable rapid, point-of-care cardiac assessment using simplified imaging protocols.

**Objectives:**

To evaluate whether a deep learning model can accurately detect significant valvular and ventricular dysfunction using only a single two-dimensional apical four-chamber echocardiographic view, including images acquired by non-cardiologists with handheld ultrasound devices.

**Methods:**

We retrospectively analyzed 120,127 echocardiographic studies from a tertiary medical center to train and validate a deep learning model for identifying moderate-or-greater mitral or tricuspid regurgitation, right ventricular dysfunction, and reduced left ventricular ejection fraction (≤40%). A prospective cohort of 209 patients underwent handheld point-of-care cardiac ultrasound performed by non-cardiologist physicians, with same-hospitalization comprehensive echocardiography as the reference standard.

**Results:**

In retrospective testing, model areas under the curve (AUCs) were 0.883 for mitral regurgitation, 0.913 for tricuspid regurgitation, 0.940 for right ventricular dysfunction, and 0.982 for reduced ejection fraction. In the prospective cohort, AUCs were 0.72, 0.87, 0.95, and 0.97 for the same respective targets.

**Conclusions:**

A single-view deep learning model demonstrated strong diagnostic accuracy for detecting significant valvular and ventricular dysfunction across both standard and handheld ultrasound acquisitions. This approach may facilitate rapid, scalable cardiac function screening by non-cardiologists in diverse clinical environments.

**Clinical Trial Registration:**

identifier NCT05455541.

## Introduction

Valvular heart disease (VHD) and heart failure (HF) are global health concerns, contributing significantly to morbidity and mortality, with a rising burden on healthcare systems, particularly in terms of diagnostic capacity (1, 2). Early and accurate diagnosis of key indicators—such as mitral regurgitation (MR), tricuspid regurgitation (TR), right ventricular dysfunction (RVD), and reduced left ventricular ejection fraction (LVEF)—can guide therapeutic decisions, enable timely interventions, and improve clinical outcomes (3, 4) These structural and functional dysfunctions often serve as early warning signs of cardiac decompensation and are closely linked with long-term prognosis. In particular, prompt identification of subclinical or asymptomatic dysfunction may delay disease progression and promote earlier treatment and timely referral for intervention (5–8).

**Table 1:** Retrospective test set—clinical and echocardiographic characteristics.

Characteristic	All (*n* = 20,960)
Age, years	66 ± 16.3 (IQR, 54–76)
Sex, male (%)	12,213 (58)
BMI, kg/m^2^	27 ± 5.2
Systolic BP, mm Hg	130 ± 22.4
LVEF, %	53.8 ± 12.0
Reduced LVEF (<41%), *n* (%)	3,553 (17)
LVEDD, cm	4.8 ± 0.70
LVESD, cm	3.2 ± 0.8
LVPWT, cm	0.9 ± 0.4
IVS, cm	1.07 ± 0.3
LVMI, g/m^2^	54.60 ± 32.20
LA-AP diameter, cm	3.9 ± 0.7
Aortic stenosis, *n* (%)	1,263 (6)
Aortic insufficiency, *n* (%)	419 (14.0)
Mitral regurgitation, *n* (%)	9,158 (44)
Tricuspid regurgitation, *n* (%)	9,271 (44)
TRSPG, mm Hg	28.50 ± 10.50
EMRAP, mm Hg	8.09 ± 3.7
Estimated sPAP, mm Hg	36.3 ± 12.9
Ischemic heart disease (%)	6,821 (33)
COPD, *n* (%)	1,100 (5)
Dyslipidemia, *n* (%)	8,977 (43)
Cerebrovascular accident, *n* (%)	2,525 (12)
Peripheral vascular disease, *n* (%)	1,142 (6)
Obesity, *n* (%)	5,890 (28)
Malignancy, *n* (%)	2,500 (12)
Smoking, *n* (%)	2,634 (13)
CKD (GFR < 60 mL/min/1.73m^2^)	3,533 (17)

BMI, body mass index; BP, blood pressure; LVEF, left ventricular ejection fraction; LVEDD, left ventricular end-diastolic diameter; LVESD, left ventricular end-systolic diameter; LVPWT, left ventricular posterior wall thickness; IVS, interventricular septum thickness; LVMI, left ventricular mass index; LA-AP, left atrial anteroposterior diameter; CKD, chronic kidney disease; GFR, glomerular filtration rate; COPD, chronic obstructive pulmonary disease; TRSPG, tricuspid regurgitation systolic pressure gradient; EMRAP, estimated mean right atrial pressure; sPAP, systolic pulmonary artery pressure.

**Table 2:** Prospective test set—clinical and echocardiographic characteristics.

Characteristic	All (*n* = 209)
Age, years	71 ± 14.3
Sex, male (%)	120 (57)
BMI, kg/m^2^	26.3 ± 5.2
Systolic BP, mm Hg	123 ± 22.1
LVEF, %	50.2 ± 12.4
Reduced LVEF (<41%), *n* (%)	51 (24)
LVEDD, cm	4.7 ± 0.7
LVESD, cm	3.2 ± 0.9
LVPWT, cm	0.9 ± 0.17
IVS, cm	1.07 ± 0.2
LVMI, g/m^2^	94.3 ± 31.3
LA-AP diameter, cm	4.4 ± 0.7
Aortic stenosis, *n* (%)	16 (7.6)
Aortic insufficiency, *n* (%)	5 (2.3)
Mitral regurgitation, *n* (%)	63 (30.1)
Tricuspid regurgitation, *n* (%)	48 (22.9)
TRSPG, mm Hg	30.9 ± 11.8
EMRAP, mm Hg	9.2 ± 4.8
Estimated sPAP, mm Hg	40.2 ± 14.5
COPD, *n* (%)	20 (9.5)
Dyslipidemia, *n* (%)	102 (48.8)
Cerebrovascular accident, *n* (%)	27 (12.9)
Peripheral vascular disease, *n* (%)	27 (12.9)
Obesity, *n* (%)	45 (21.5)
Malignancy, *n* (%)	7 (3.34)
Smoking, *n* (%)	57 (27.2)
CKD (GFR < 60 mL/min/1.73m^2)^	39 (18.6)

BMI, body mass index; BP, blood pressure; LVEF, left ventricular ejection fraction; LVEDD, left ventricular end-diastolic diameter; LVESD, left ventricular end-systolic diameter; LVPWT, left ventricular posterior wall thickness; IVS, interventricular septum thickness; LVMI, left ventricular mass index; LA-AP, left atrial anteroposterior diameter; CKD, chronic kidney disease; GFR, glomerular filtration rate; COPD, chronic obstructive pulmonary disease; TRSPG, tricuspid regurgitation systolic pressure gradient; EMRAP, estimated mean right atrial pressure; sPAP, systolic pulmonary artery pressure.

Despite the clinical importance of early diagnosis, significant practical challenges in reliably identifying these cardiac conditions remain across diverse care settings. While transthoracic echocardiography (TTE) is the gold standard for evaluating valvular and ventricular function, it typically requires comprehensive multi-view imaging, expert interpretation by trained cardiologists, and significant infrastructure (9). This creates a bottleneck, especially in resource-limited environments or during off-hours. Cardiac auscultation and physical examination, though widely taught and routinely used, have limited sensitivity and specificity, reducing their reliability in detecting pathological murmurs or chamber dysfunction (10).

The increasing demand for echocardiography in both inpatient and outpatient settings has exposed a gap between diagnostic need and available resources. In rural regions, where cardiovascular mortality is disproportionately high, access to cardiologists and echocardiography is often scarce (11). These limitations can contribute to health disparities, as underserved populations—particularly those in rural or resource-limited settings—may be less likely to receive timely cardiac imaging and intervention. While handheld devices and point-of-care ultrasound (FoCUS) offer potential solutions, effective use still requires training in both image acquisition and interpretation, which can be challenging to standardize and scale (12).

To address these limitations, the purpose of this study is to develop and validate a deep learning model capable of detecting clinically significant MR, TR, RVD, and reduced LVEF using only a single apical 4-chamber (A4C) view from transthoracic echocardiography. Unlike traditional workflows that rely on full studies and expert interpretation, our approach aims to enable focused, automated bedside assessment by non-cardiologists using handheld ultrasound devices. By automating the interpretation of key diagnostic features from a single view, we seek to create a solution that is scalable, time-efficient, and accessible across clinical environments—including outpatient clinics, emergency departments, and underserved settings—where timely echocardiographic evaluation may otherwise be unavailable.

## Materials and methods

### Study population and retrospective data source

This study incorporated two distinct cohorts: a retrospective transthoracic echocardiography (TTE) cohort and a prospective point-of-care FoCUS cohort. The retrospective dataset comprised adult patients (aged ≥18 years) who underwent TTE at Sheba Medical Center between 2007 and 2022. Data were extracted from the SHEBAHEART big data registry, a comprehensive institutional database linking echocardiographic studies with clinical information. Sheba Medical Center is Israel's largest medical center, conducting over 120,000 annual admissions and approximately 22,000 echocardiographic exams per year. Echocardiographic data and corresponding electronic health records were used to develop and validate the deep learning model. The study protocol received Institutional Review Board approval (Sheba IRB), and individual consent was waived due to retrospective de-identification. Following quality control and view classification procedures, 28,790 exams were excluded due to suboptimal views or data corruption, and 1,887 were removed as duplicates. The final cohort included 120,127 unique adult patients, representing a diverse clinical population and encompassing both inpatient and outpatient exams performed across 20 different ultrasound device models. Given the large cohort size (>120,000), no formal sample size calculation was performed. The dataset was deemed sufficient to support model development, stratification, and robust internal validation. Missing data on outcomes or image labels were excluded from model development. No imputation was performed, and only complete studies with fully labeled 2D apical 4-chamber views were used for training and evaluation. Each echocardiographic study included 20–80 still images and video loops, acquired following standardized acquisition protocols. Information on treatments received was not included in the model and was not used for training or evaluation. This dataset was randomly divided into training (*n* = 76,342; 62%), validation (*n* = 22,825; 19%), and test (*n* = 20,960; 17%) subsets.

### Prospective FoCUS study design and implementation

The prospective FoCUS validation cohort (*n* = 209) was derived from a single-arm, single-center clinical study conducted between 2022 and 2023 at Sheba Medical Center (ClinicalTrials.gov identifier NCT05455541) that was previously presented (13). It included adult patients presenting to the emergency department or admitted to internal medicine wards without recent formal echocardiography, or patients with complex structural heart disease or prosthetic valves. Participants verbally consented to AI-enhanced FoCUS evaluation as part of their diagnostic assessment. Internal and emergency medicine residents performed bedside FoCUS exams without prior echocardiographic training. Residents completed a structured six-hour hands-on workshop on 2D and color Doppler acquisition, supervised by expert cardiologists and sonographers. Using Philips Lumify S4-1 handheld ultrasound devices connected to Samsung tablets and integrated with FDA-cleared Aisap.ai software, residents acquired focused images from four standard views (parasternal long- 2D and color Doppler, apical 4-chamber -2D and color Doppler, and subcostal for IVC assessment). Studies were intended for rapid bedside screening rather than comprehensive echocardiography; tissue Doppler imaging (TDI), pulsed-wave (PW), and continuous-wave (CW) Doppler were not performed. The model requires adequate apical 4-chamber image quality for reliable output. The AI platform provided real-time feedback on image quality and diagnostic value, helping users improve acquisition in real-time. The system displayed a quantitative image quality indicator (expressed as a percentage) before image capture, guiding users to optimize probe positioning and ensuring consistent acquisition of diagnostically adequate images across operators. A cardiologist reviewed and approved each physician's first 20 scans before autonomous participation. A support team, including a cardiologist, sonographer, and technician, was available throughout the study. Residents performed AI-enhanced FoCUS exams in response to a clinical indication or as part of routine physical assessment. Image data and AI-generated interpretations were saved to a secure cloud platform and incorporated into the electronic medical record (EMR). A formal echocardiogram was performed by certified sonographers and interpreted by blinded cardiologists for patients with suspected clinically significant findings. The median time between FoCUS and formal TTE was 20 h [IQR: 14–37 h]. The sample size for the prospective FoCUS cohort (*n* = 209) was determined pragmatically based on feasibility within the clinical study window. While formal power calculations were not performed, this cohort size was sufficient for initial external validation across key diagnostic categories. No formal protocol was prepared for this analysis.

### AI model development and training

The deep learning model used in this study was developed collaboratively by AiSAP.ai (Ramat-Gan, Israel) and Sheba Medical Center and was previously described (14). The full infrastructure of this technology (AISAP CARDIO V1.0), originally validated for parasternal, sub-costal, as well as apical views, is FDA-approved as a software platform that automatically processes and analyzes acquired cardiac FoCUS images. The model was trained using 120,127 TTE studies, each annotated by board-certified cardiologists following ASE guidelines (9). The training data included over 250,000 echocardiographic loops collected from 2009 to 2020 using diverse machines and clinical protocols. Each exam provided 20–80 clips from standard views. Outcomes used for model training and evaluation were extracted from clinical echocardiographic reports created independently by cardiologists who were blinded to model predictions. The dataset was randomly split into training (76,342; 62%), validation (22,825; 19%), and test (20,960; 17%) cohorts. Internal validation was conducted using the independent validation set during training, with model selection based on performance metrics (AUC and RMSE) on this set. The apical 4-chamber (A4C) grayscale view, without the use of color Doppler, was chosen as the sole input modality due to its widespread use, standardized acquisition, and ability to simultaneously visualize both ventricles and the atrioventricular valves. No additional clinical or imaging predictors were included. The model architecture employed a combination of EfficientNetV2 convolutional neural networks and multi-head self-attention transformers. Ten frames per clip were sampled over 1.5-s windows to capture full cardiac cycles. Each frame was converted into a feature vector, aggregated via transformer layers, and processed through regression or classification heads to estimate continuous (e.g., LVEF) or categorical (e.g., valvular severity) outputs. Model training was optimized using L2-squared error loss for regression and cross-entropy loss for classification. Image preprocessing and quality control procedures were applied uniformly across all patient subgroups and acquisition devices. No subgroup-specific adjustments were made during data filtering or preparation. Patients or members of the public were not involved in the design, conduct, or reporting of this study. All echocardiographic studies were exported in DICOM format, anonymized, and automatically segmented by view classification networks before inclusion. Frames were uniformly resized and normalized to standard pixel intensity ranges before being input to the model. Internal validation was performed on a hold-out dataset with no patient overlap, ensuring that training and testing were fully independent. All analyses followed a prespecified pipeline and were repeated twice by separate analysts to confirm reproducibility.

### Definition of significant valvular and ventricular dysfunction

All echocardiographic reports used as the reference (“ground truth”) were interpreted by board-certified cardiologists at Sheba Medical Center with advanced expertise in echocardiography, in accordance with the American Society of Echocardiography (ASE) guidelines (15). Significant regurgitation was defined as at least moderate mitral and/or tricuspid regurgitation as assessed by ASE Significant left ventricular dysfunction was defined by an LVEF of 40% or less. Significant right ventricular dysfunction was defined as at least moderate global right ventricular dysfunction. Reference classifications for MR, TR, LVEF, and RV function were based on standard qualitative and quantitative parameters, including fractional area change, TAPSE, and color Doppler findings when available. These cardiologist-derived assessments served as the supervised reference (“ground truth”) labels for model training, enabling the algorithm to learn grayscale motion and structural patterns that correlate with multi-parametric Doppler-based findings. Although the AI model analyzed only grayscale apical four-chamber images, it was trained on Doppler-labeled studies, enabling recognition of visual patterns associated with significant valvular and ventricular dysfunction.

### Statistical analysis

In the retrospective cohort, the diagnostic performance of the AI model based on a single apical 4-chamber view was evaluated against the comprehensive formal transthoracic echocardiographic (TTE) report generated from the same examination. In the prospective point-of-care cohort, FoCUS-derived single-view findings were compared to a formal comprehensive TTE examination performed during the same hospital visit. Continuous variables were assessed using Pearson correlation to evaluate the linear relationship between AI-derived left ventricular ejection fraction (LVEF) estimates and standard measurements, with agreement further quantified by root mean square error (RMSE) and mean absolute error (MAE). For categorical analyses, binary thresholds were applied: the reduced left ventricular function was defined as LVEF <40% and moderate-or-greater severity was defined for right ventricular (RV) systolic dysfunction, mitral regurgitation (MR), and tricuspid regurgitation (TR). Diagnostic performance metrics were calculated from confusion matrices, including sensitivity, specificity, positive predictive value (PPV), negative predictive value (NPV), and accuracy. As The model was designed as a qualitative screening tool to classify reduced EF (<40%) rather than to estimate precise numerical values, categorical performance metrics (AUC, sensitivity, specificity, and RMSE) were used. Statistical significance was defined as a two-tailed *p*-value <0.05. Fairness analyses across sociodemographic subgroups were not performed in this study, as the primary objective was to evaluate overall model performance across diverse imaging conditions. All statistical analyses were conducted using R software (version 4.4.3, R Foundation for Statistical Computing). This manuscript was prepared in accordance with the TRIPOD + AI reporting guideline for studies developing or validating prediction models using machine learning methods (16).

## Results

### Retrospective TTE validation cohort

#### Patient characteristics

The retrospective validation cohort consisted of 20,960 unique adult patients with a median age of 66 years (IQR: 54–76), of whom 58% (*N* = 12,213) were male (Table 1). The average body mass index (BMI) was 27 ± 5.2 kg/m^2^, and the mean systolic blood pressure at the time of the exam was 130 ± 22 mmHg. The mean left ventricular ejection fraction (LVEF) was 54 ± 12% (*N* = 20,960), with 17% (*n* = 3,553) of patients showing reduced LVEF (<40%). The left ventricular end-diastolic and end-systolic diameters (LVEDD and LVESD) were 48 ± 7 mm and 32 ± 9 mm, respectively. Mean posterior wall thickness and interventricular septum thickness were 10 ± 4 mm and 11 ± 3 mm, respectively. The estimated left ventricular mass index (LVMI) was 55 ± 32 g/m^2^, and the left atrial anteroposterior diameter measured 4 ± 0.8 cm. Significant valvular disease was not uncommon. There were 9,158 (44%) patients with mitral regurgitation (MR), 9,271 (44%) with tricuspid regurgitation (TR), 1,263 (6%) with significant aortic stenosis, and 4,143 (14%) with aortic insufficiency. Right-sided pressures were elevated in some patients, with estimated systolic pulmonary artery pressure (sPAP) of 36 ± 13 mmHg.

Documented comorbidities included obesity in 5,890 (28%) patients, chronic kidney disease (CKD) with GFR <60 mL/min/1.73m^2^ in 3,533 (17%) patients, ischemic heart disease in 6,821 (33%) patients, history of cerebrovascular accident in 2,525 (12%) patients, peripheral vascular disease in 1,142 (6%), chronic obstructive pulmonary disease in 1,100 (5%) patients, malignancy of any type in 2,500 (12%) patients, and a history of or active smoking in 2,634 (13%) patients.

### Performance of the AI model

**Figure 1:**
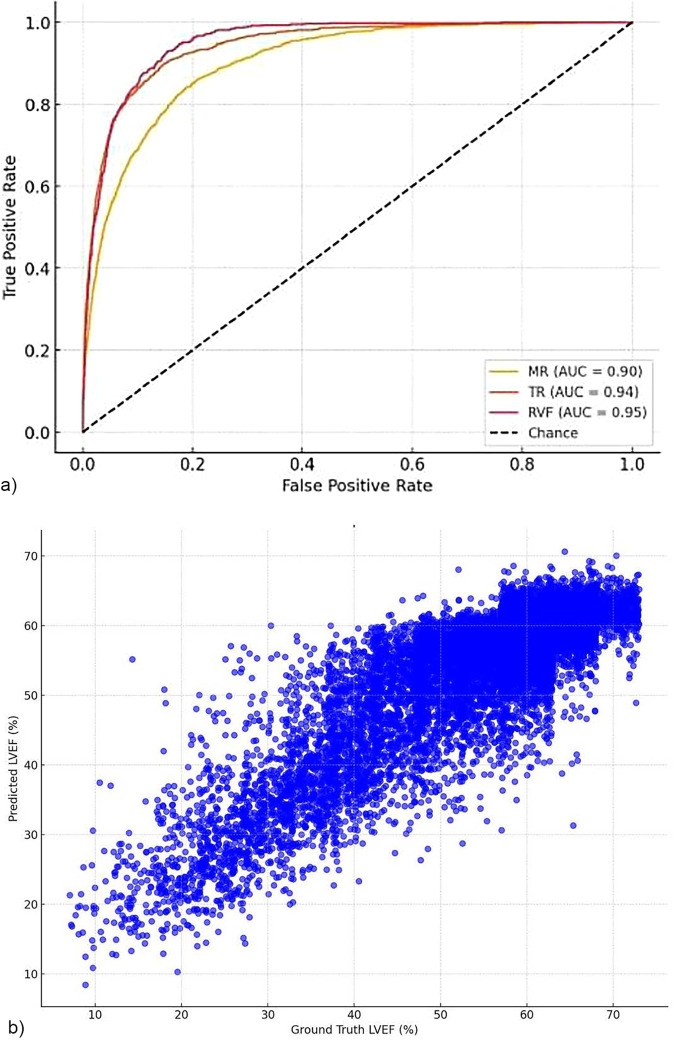
**(a)** Area under the curve (AUC) for single-view detection of significant. Cardiac Dysfunction in a Retrospective Echocardiography Cohort. Receiver-operating-characteristic curves for the detection of moderate grade or higher mitral regurgitation (MR), tricuspid regurgitation (TR), and right ventricular dysfunction (RVF) using a single apical four-chamber view. The area under the curve (AUC) was 0.90 for MR, 0.94 for TR, and 0.95 for RVF. The diagonal dashed line indicates the line of no discrimination. (b) Scatter plot of AI-estimated versus in retrospective echocardiograms. Scatter plot illustrating the relationship between AI-predicted left ventricular ejection fraction (LVEF) and the groud truth measurements derived from comprehensive transthoracic echocardiagraphy in the retrospective validation dateset (N = 20,960). Each point represents a single patient examinstion.

The AI model, evaluated using a single apical 4-chamber grayscale echocardiographic view, showed good diagnostic accuracy in this large and diverse clinical cohort (Figures 1a,b). For moderate or greater MR detection, the model achieved an AUC of 0.883, with 80% accuracy, 74.4% sensitivity, and 84.4% specificity. TR detection yielded an AUC of 0.913, with 83.5% accuracy, 76.6% sensitivity, and 88.7% specificity. Right ventricular dysfunction was identified with an AUC of 0.94, sensitivity of 75.7%, and specificity of 93.9%.

For LVEF prediction, the model returned a root mean square error (RMSE) of 4.78 percentage points and a mean absolute error (MAE) of 3.34 percentage points. In binary identifying patients with reduced LVEF (<41%), the model achieved 94.2% accuracy, a sensitivity of 70.7%, a specificity of 99.0% and an AUC of 0.982.

### Prospective FoCUS cohort

#### Patient characteristics and recruitment

The prospective cohort included 209 patients from a larger sample of 660 enrolled consecutively between June 2022 and June 2023 (Table 2). All patients were scanned using handheld ultrasound devices and mobile tablet screen sets by 13 internal medicine residents. Each resident completed a mean of 50 examinations (range: 8–158; median: 29). The average scan duration was 7 min and 20 s (range: 1.5–13 min). The mean age of patients in the FoCUS cohort was 71 ± 14 years, of whom 120 (57%) were male. The mean BMI was 26 ± 5 kg/m^2^, and the mean systolic blood pressure at admission was 123 ± 22 mmHg. The average LVEF was 50 ± 12%, with 51 (24%) patients classified as having reduced LVEF. There were 63 (30%) and 48 (23%) patients with significant MR and TR, respectively. Structural and functional measurements included LVEDD of 47 ± 7 mm, LVESD of 33 ± 9 mm, posterior wall thickness of 95 ± 17 mm, interventricular septum thickness of 11 ± 3 mm, estimated LVMI of 94.35 ± 31.32 g/m^2^, and left atrial diameter of 45 ± 7 mm. Estimated sPAP was 40 ± 15 mmHg. Relevant clinical comorbidities in the FoCUS cohort included dyslipidemia in 48.8% (*n* = 102), CKD of any degree in 18.6% (*n* = 39), diabetes mellitus in 28% (*n* = 58), hypertension in 48% (*n* = 100), ischemic heart disease in 32% (*n* = 67), atrial fibrillation in 18% (*n* = 38), and pre-existing heart failure in 15% (*n* = 31). Six percent (*n* = 12) had a known history of valvular heart disease. Past or active smoking was documented in 27.2% (*n* = 57). Common presenting complaints were chest pain in 31% (*n* = 65), dyspnea in 22% (*n* = 46), malaise in 10% (*n* = 21), fever in 10% (*n* = 21), and syncope in 6% (*n* = 13).

### Performance of the AI model

**Figure 2:**
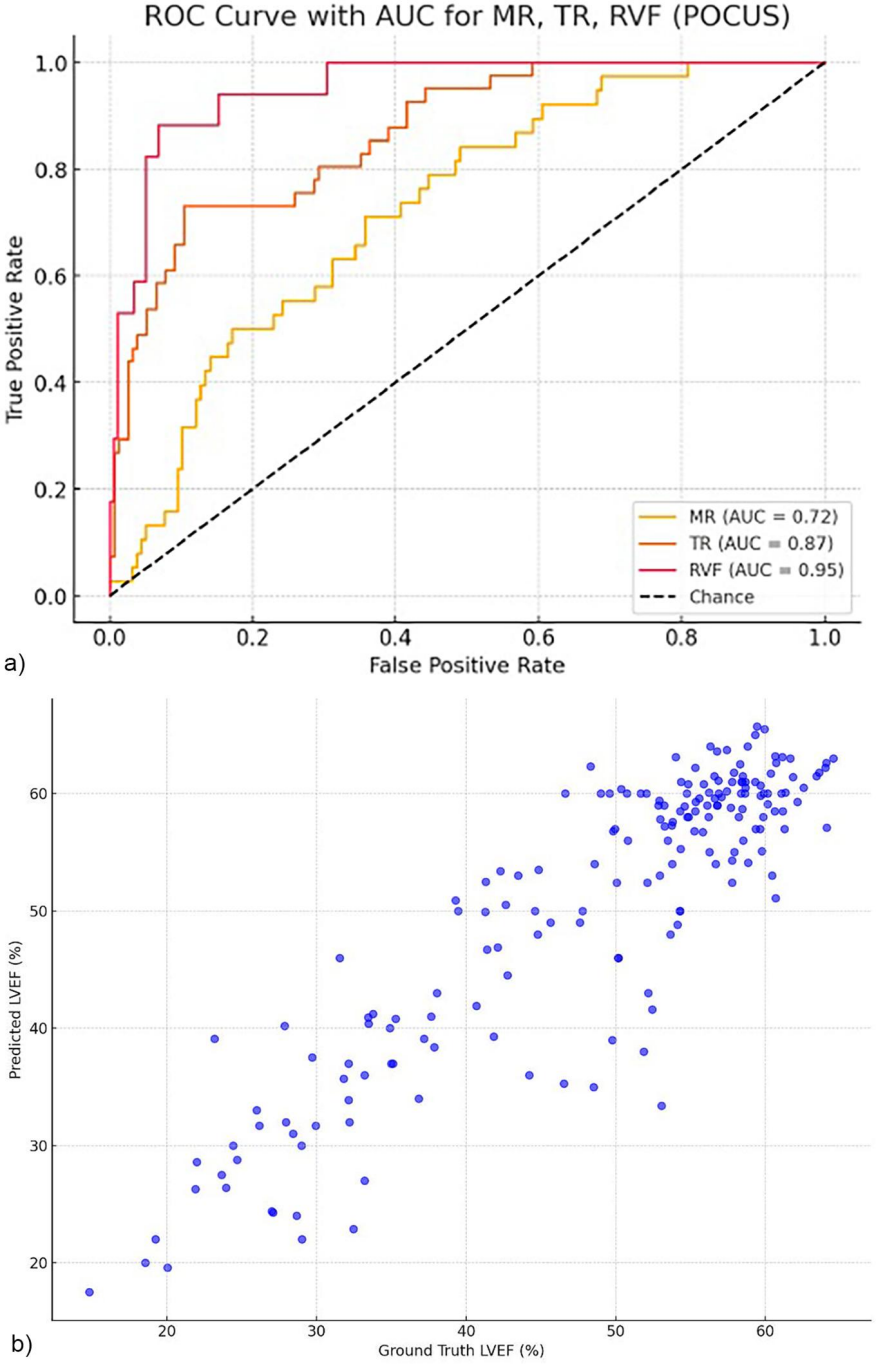
**(a)** Area under the curve (AUC) for single-view detection of significant cardiac dysfunction in a prospective FOCUS cohort. Receiver-operating-characteristic curves for the detection of moderate grade or higher mitral regurgitation (MR), tricuspid regurgitation (TR), and right ventricular dysfunction (RVF) in the handheld point-of-care ultrasound (FOCUS ) cohort. The area under the curve (AUC) was 0.72 for MR, 0.87 for TR, and 0.95 for RVF. The diagonal dashed line indicates the line of no discrimination. **(b)** Scatter plot comparing Al-predicted left ventricular ejection fraction (LVEF) and reference standard echocardiographic measurements in the prospective FoCUS validation cohort (*n* = 209). Scatter plot comparing Al-predicted left ventricular ejection fraction (LVEF) estimates with reference standard echocardiographic measurements in the prospective point-of-care ultrasound (FoCUS) validation cohort (*N* = 209). FoCUS examinations were acquired by non-cardiologist physicians using handheld ultrasound devices.

In this focused point-of-care cardiac ultrasound cohort, the AI model demonstrated consistent diagnostic performance (Figures 2a,b). To detect moderate or greater MR, the model achieved an AUC of 0.72, with an accuracy of 64%, sensitivity of 71.1% and a specificity of 61.2%. For TR, the AUC was 0.8674, with an accuracy of 83.7%, 73.2% sensitivity, and 87.0% specificity. Right ventricular dysfunction was detected with an AUC of 0.9521, sensitivity of 94.1%, and specificity of 82.0%. Predicting reduced LVEF (<41%) in the FoCUS cohort resulted in 78.3% sensitivity and 91.5% specificity, an AUC of 0.970 with a Pearson correlation coefficient of 0.83 compared to standard echocardiographic values.

## Discussion

This study demonstrated that a deep learning model can accurately detect significant valvular and ventricular dysfunction using only a single two-dimensional apical 4-chamber echocardiographic view. Importantly, this performance was confirmed in formal TTEs and real-world FoCUS acquisitions performed by non-cardiologist physicians in clinical practice. The ability to extract clinically meaningful diagnostic information from a single limited view holds significant implications for the future of cardiac patient assessment at the bedside by non-cardiologists.

The model presented in this study achieved good diagnostic accuracy for moderate-or-greater MR, TR, and right ventricular dysfunction. In the FoCUS cohort, where image quality and acquisition conditions were less controlled, performance remained comparable. In the FoCUS cohort, model performance for MR detection was lower (AUCs of 0.7248 for MR) than in the formal TTE cohort and lower than for TR detection, likely reflecting the dynamic nature of mitral regurgitation (17). MR severity can fluctuate with changes in loading conditions, ischemia, and treatment response (18), making it a challenging target for single-view, real-world assessments.

The proposed clinical use of this technology is as a decision-support tool for non-cardiologist clinicians performing point-of-care cardiac ultrasound in acute or resource-limited settings. By providing automated feedback on major valvular or ventricular dysfunction from a single view, it facilitates early identification and triage of patients requiring comprehensive echocardiographic evaluation or cardiology referral.

Since the tested approach is novel, comparisons to the results of other groups are naturally limited. However, several relevant reference models are pertinent to our study. Vrudhula et al. (19), who developed EchoNet-TR, a deep learning pipeline for detecting TR based on 2D and Doppler apical 4-chamber views from TTE studies. The authors achieved AUCs of 0.928–0.951 for moderate or severe TR. Our model achieved an AUC of 0.913 on TTE clips and 0.8674 on FoCUS clips, using only a single apical 4-chamber view. EchoNet-TR required color Doppler, potentially limiting applicability in point-of-care settings. In contrast, our model relies only on B-mode imaging, making it more suitable for personnel with limited training and ability and independent of the device capabilities being used.

The concept that valvular dysfunctions can be assessed from a single two-dimensional view has also been supported by Holste et al., who demonstrated that deep neural networks (DNNs) can reliably diagnose severe aortic stenosis (AS) using parasternal long-axis videos without Doppler imaging (20). Their findings highlight how DNNs can extract complex structural and functional information from grayscale B-mode imaging, identifying features such as valve thickening, reduced leaflet mobility, and chamber remodeling. Similarly, our study shows that significant valvular disease can be accurately assessed without color Doppler, broadening the applicability of AI-assisted FoCUS in real-world clinical practice. These observations suggest deep learning models can capture high-dimensional physiological information embedded within standard 2D echocardiographic images. The deep learning framework used in this study has previously demonstrated its versatility in analyzing echocardiographic images beyond conventional diagnostic tasks; Notably, the same architecture has been shown to accurately estimate patient age and sex from standard transthoracic echocardiographic views (4). This notion is also supported by a study by Malins et al., which showed that AI can accurately estimate left ventricular ejection fraction from a single 2D echocardiographic frame without requiring full video clips (20). Similarly, our results show that significant valvular dysfunction and ventricular performance can be reliably assessed from a single apical 4-chamber view, supporting the broader potential of deep learning to extract diagnostic information from limited ultrasound imaging. More recently, Holste et al. also reported the development and validation of PanEcho, a comprehensive multitask deep learning system capable of automating echocardiogram interpretation across 39 labels and measurements, achieving a median AUC of 0.91 and demonstrating robust performance even in limited imaging protocols and point-of-care acquisitions (21). This notion is also supported by a study by Malins et al., which demonstrated that AI can accurately estimate left ventricular ejection fraction from a single 2D echocardiographic frame without requiring full video clips (22). These findings highlight the growing evidence that AI can perform near-complete echocardiographic analysis with high accuracy and that simplified acquisition protocols may suffice for many diagnostic tasks when paired with advanced neural networks.

Windows our results extend earlier findings by demonstrating that left ventricular systolic function, significant valvular lesions, and right ventricular dysfunction can be reliably inferred from a single limited view. Traditional echocardiography relies on acquiring multiple standardized views, requiring skilled operators and substantial time (23). By enabling accurate diagnostic assessments from a single apical 4-chamber view, AI could simplify workflows, reduce examination time, and expand access to echocardiographic evaluation in settings where expert sonographers are unavailable. Integrating such algorithms into handheld devices may enable non-specialists to perform focused cardiac evaluations, facilitating earlier diagnosis and treatment of heart failure and valvular disease at the point of care.

Based on our findings, the AI-assisted handheld ultrasound model may eventually serve as an effective screening tool for valvular and ventricular dysfunction. It meets key Wilson and Jungner criteria (24): the target conditions are common, serious, and treatable; the model detects early dysfunction from a single apical 4-chamber view; and the test is simple, acceptable, and scalable. Deployment on handheld devices enables low-cost, repeatable case-finding in settings with limited access to formal echocardiography.

Several limitations of this study should be acknowledged. First, although the retrospective cohort was large and heterogeneous, the prospective FoCUS cohort was relatively small, derived from a single healthcare system, and included patients whose symptoms prompted advanced cardiac assessment. This may introduce selection bias and limit generalizability. Second, the study focused exclusively on apical 4-chamber views, which can be more technically challenging to acquire, particularly for personnel with basic training (25). Optimizing apical views may require careful patient positioning, breath control, and probe angulation, skills that may not be consistently available in all clinical settings. Furthermore, apical imaging windows are more susceptible to suboptimal quality due to body habits and typically require transducers with sufficient penetration power, which may not always be available on handheld or pocket-sized ultrasound devices; this was addressed by the integrated feedback application for assisting in image quality improvement. Although suboptimal images were excluded during training, the model performed strongly in the prospective FoCUS cohort of handheld scans by non-expert physicians, supporting real-world robustness. The large, diverse datasets used reduce potential performance variability. Additionally, our model does not assess the aortic valve, as the apical 4-chamber view does not provide adequate visualization of aortic valve morphology or function. As the model relies on a single grayscale apical four-chamber view, it excludes Doppler and multi-view data—supporting point-of-care use but limiting accuracy for dynamic lesions such as mitral regurgitation and reducing interpretability for clinicians. Given the variability and limited reliability of current explainability approaches for dynamic echocardiographic video data—and the clinically focused nature of this study—we did not incorporate these methods, and this is acknowledged as a limitation. The complex temporal interplay of features across cardiac cycles further contributes to the model's ‘black-box’ nature.

Beyond simply evaluating model performance, our findings emphasize the clinical value of utilizing a single limited view to trigger real-time alerts for cardiac abnormalities. Instead of intending to fully replace comprehensive echocardiography, the model's primary role is to function as an early warning system, prompting further evaluation and optimizing patient triage.

## Conclusions and clinical implications

We introduce a deep learning model capable of accurately assessing significant valvular dysfunction, right ventricular dysfunction, and left ventricular ejection fraction from a single apical 4-chamber echocardiographic view. Our model demonstrated strong diagnostic performance across TTE datasets and real-world FoCUS studies. While further validation is needed, this approach could significantly expand access to cardiac screening, simplify clinical workflows, and enable rapid, AI-assisted assessment of cardiac function at the point of care.

## Data Availability

The raw data supporting the conclusions of this article will be made available by the authors, without undue reservation.

## References

[1] Coffey S Roberts-Thomson R Brown A Carapetis J Chen M Enriquez-Sarano M Global epidemiology of valvular heart disease. Nat Rev Cardiol. (2021) 18(12):853–64. 10.1038/s41569-021-00570-z 34172950

[2] Docherty KF Lam CSP Rakisheva A Coats AJS Greenhalgh T Metra M Heart failure diagnosis in the general community—who, how and when? A clinical consensus statement of the Heart Failure Association (HFA) of the European Society of Cardiology (ESC). Eur J Heart Fail. (2023) 25(8):1185–98. 10.1002/ejhf.2946 37368511

[3] Sengupta PP Kluin J Lee SP Oh JK Smits AIPM. The future of valvular heart disease assessment and therapy. Lancet. (2024) 403(10436):1590–602. 10.1016/S0140-6736(23)02754-X 38554727

[4] Dini FL Pugliese NR Ameri P Attanasio U Badagliacca R Correale M Right ventricular failure in left heart disease: from pathophysiology to clinical manifestations and prognosis. Heart Fail Rev. (2023) 28(4):757–66. 10.1007/s10741-022-10282-2 36284079 PMC9596338

[5] Messika-Zeitoun D Baumgartner H Burwash IG Vahanian A Bax J Pibarot P Unmet needs in valvular heart disease. Eur Heart J. (2023) 44(21):1862–73. 10.1093/eurheartj/ehad121 36924203

[6] Maddox TM Januzzi JL Allen LA Breathett K Brouse S Butler J 2024 ACC expert consensus decision pathway for treatment of heart failure with reduced ejection fraction: a report of the American College of Cardiology solution set oversight committee. J Am Coll Cardiol. (2024) 83(15):1444–88. 38466244 10.1016/j.jacc.2023.12.024

[7] Nishimura RA O’Gara PT Bavaria JE Brindis RG Carroll JD Kavinsky CJ 2019 AATS/ACC/ASE/SCAI/STS expert consensus systems of care document: a proposal to optimize care for patients with valvular heart disease: a joint report of the American Association for Thoracic Surgery, American College of Cardiology, American Society of Echocardiography, Society for Cardiovascular Angiography and Interventions, and Society of Thoracic Surgeons. J Am Coll Cardiol. (2019) 73(20):2609–35. 10.1016/j.jacc.2018.10.007 31010719

[8] Angaran P Dorian P Ha ACT Thavendiranathan P Tsang W Leong-Poi H Association of left ventricular ejection fraction with mortality and hospitalizations. J Am Soc Echocardiogr. (2020) 33(7):802–11.e6. 10.1016/j.echo.2019.12.016 32164977

[9] Mitchell C Rahko PS Blauwet LA Canaday B Finstuen JA Foster MC Guidelines for performing a comprehensive transthoracic echocardiographic examination in adults: recommendations from the American Society of Echocardiography. J Am Soc Echocardiogr. (2019) 32(1):1–64. 10.1016/j.echo.2018.06.004 30282592

[10] Davidsen AH Andersen S Halvorsen PA Schirmer H Reierth E Melbye H. Diagnostic accuracy of heart auscultation for detecting valve disease: a systematic review. BMJ Open. (2023) 13(3):e068121. 10.1136/bmjopen-2022-068121 36963797 PMC10040065

[11] Harrington RA Califf RM Balamurugan A Brown N Benjamin RM Braund WE Call to action: rural health: a presidential advisory from the American Heart Association and American Stroke Association. Circulation 2020;141(10):E615–44. 10.1161/CIR.0000000000000753 32078375

[12] Zisis G Yang Y Huynh Q Whitmore K Lay M Wright L Nurse-provided lung and inferior vena cava assessment in patients with heart failure. J Am Coll Cardiol. (2022) 80(5):513–23. 10.1016/j.jacc.2022.04.064 35902175

[13] Fisher L Yarkoni Y Faierstein K Fiman M Segal E Rubin N Enhancing handheld point-of-care echocardiography with artificial intelligence: a prospective clinical trial. J Am Coll Cardiol. (2024) 83(13):2344.

[14] Faierstein K Fiman M Loutati R Rubin N Manor U Am-Shalom A Artificial intelligence assessment of biological age from transthoracic echocardiography: discrepancies with chronologic age predict significant excess mortality. J Am Soc Echocardiogr. (2024) 37(8):725–35. 10.1016/j.echo.2024.04.017 38740271

[15] Zoghbi WA Adams D Bonow RO Enriquez-Sarano M Foster E Grayburn PA Recommendations for noninvasive evaluation of native valvular regurgitation: a report from the American society of echocardiography developed in collaboration with the society for cardiovascular magnetic resonance. J Am Soc Echocardiogr. (2017) 30(4):303–71. 10.1016/j.echo.2017.01.007 28314623

[16] Collins GS Moons KGM Dhiman P Riley RD Beam AL Van Calster B TRIPOD + AI statement: updated guidance for reporting clinical prediction models that use regression or machine learning methods. Br Med J. (2024) 385:e078378. 10.1136/bmj-2023-078378 38626948 PMC11019967

[17] Lancellotti P Fattouch K La Canna G. Therapeutic decision-making for patients with fluctuating mitral regurgitation. Nat Rev Cardiol. (2015) 12(4):212–9. 10.1038/nrcardio.2015.16 25666403

[18] Kubo S Kawase Y Hata R Maruo T Tada T Kadota K. Dynamic severe mitral regurgitation on hospital arrival as prognostic predictor in patients hospitalized for acute decompensated heart failure. Int J Cardiol. (2018) 273:177–82. 10.1016/j.ijcard.2018.09.093 30274752

[19] Vrudhula A Vukadinovic M Haeffele C Kwan AC Berman D Liang D Automated deep learning phenotyping of tricuspid regurgitation in echocardiography. JAMA Cardiol. (2025) 10(6):595–602. 10.1001/jamacardio.2025.0498 40238103 PMC12004246

[20] Holste G Oikonomou EK Mortazavi BJ Coppi A Faridi KF Miller EJ Severe aortic stenosis detection by deep learning applied to echocardiography. Eur Heart J. (2023) 44(43):4592–604. 10.1093/eurheartj/ehad456 37611002 PMC11004929

[21] Holste G Oikonomou EK Tokodi M Kovács A Wang Z Khera R. Complete AI-enabled echocardiography interpretation with multitask deep learning. JAMA. (2025) 334:306. 10.1001/jama.2025.8731 40549400 PMC12186137

[22] Malins JG Anisuzzaman DM Jackson JI Lee E Naser JA Rostami B Snapshot artificial intelligence—determination of ejection fraction from a single frame still image: a multi-institutional, retrospective model development and validation study. Lancet Digit Health. (2025) 7(4):e255–63. 10.1016/j.landig.2025.02.003 40148009

[23] Popescu (Chair) BA Stefanidis A Fox KF Cosyns B Delgado V Di Salvo GD Training, competence, and quality improvement in echocardiography: the European association of cardiovascular imaging recommendations: update 2020. Eur Heart J Cardiovasc Imaging. (2020) 21(12):1305–19. 10.1093/ehjci/jeaa266 33245758

[24] World Health Organization. Screening Programmes: A Short Guide. Vol. 1. Geneva: WHO Press (2020). p. 1–70. Available online at: https://apps.who.int (Accessed June 17, 2025).

[25] Spencer KT Kimura BJ Korcarz CE Pellikka PA Rahko PS Siegel RJ. Focused cardiac ultrasound: recommendations from the American society of echocardiography. J Am Soc Echocardiogr. (2013) 26(6):567–81. 10.1016/j.echo.2013.04.001 23711341

