# Correction: Artificial intelligence assessment of valvular disease and ventricular function by a single echocardiography view

**DOI:** 10.3389/fdgth.2026.1815054

**Published:** 2026-03-09

**Authors:** Lior Fisher, Michael Fiman, Ella Segal, Shira Lidar, Noa Rubin, Adiel Am-Shalom, Ido Cohen, Kobi Faierstein, Avishai M. Tsur, Ehud Schwammenthal, Robert Klempfner, Eyal Zimlichman, Ehud Raanani, Elad Maor

**Affiliations:** 1 Sheba Medical Center, Leviev Cardiovascular Institute, Ramat Gan, Israel; 2 Sheba Medical Center, Tel- Aviv Faculty of Medicine, Tel Aviv University, Tel Aviv, Israel; 3 Department of Military Medicine, Faculty of Medicine, Hebrew University of Jerusalem, Jerusalem, Israel; 4 School of Public Health, Gray Faculty of Medical & Health Sciences, Tel Aviv University, Tel Aviv, Israel

**Keywords:** artificial intelligence, heart failure, echocardiography, point-of-care imaging, valvular heart disease

Authors Michael Fiman, Noa Rubin, and Adiel Am-Shalom were erroneously assigned to Department of Military Medicine, Faculty of Medicine, Hebrew University of Jerusalem, Jerusalem, Israel. This affiliation has now been removed for Michael Fiman, Noa Rubin, and Adiel Am-Shalom.

Affiliation Sheba Medical Center, Leviev Cardiovascular Institute, Ramat Gan, Israel was omitted for authors Michael Fiman, Noa Rubin, and Adiel Am-Shalom. This affiliation has now been added for Michael Fiman, Noa Rubin, and Adiel Am-Shalom.

The original version of this article has been updated.

